# Gene Editing, Identity and Benefit

**DOI:** 10.1093/pq/pqab029

**Published:** 2021-06-05

**Authors:** Thomas Douglas, Katrien Devolder

**Affiliations:** University of Oxford, UK; University of Oxford, UK

**Keywords:** CRISPR, genome editing, genetic selection, the non-identity problem, counterfactuals, pre-emption

## Abstract

Some suggest that gene editing human embryos to prevent genetic disorders will be in one respect morally preferable to using genetic selection for the same purpose: gene editing will benefit particular future persons, while genetic selection would merely replace them. We first construct the most plausible defence of this suggestion—the benefit argument—and defend it against a possible objection. We then advance another objection: the benefit argument succeeds only when restricted to cases in which the gene-edited child would have been brought into existence even if gene editing had not been employed. Our argument relies on a standard account of comparative benefit which has recently been criticised on the grounds that it succumbs to the so-called ‘pre-emption problem’. We end by considering how our argument would be affected were the standard account revised in an attempt to evade this problem. We consider three revised accounts and argue that, on all three, our critique of the benefit argument stands.

## I. THE BENEFIT ARGUMENT

*Edited Larry.* Lesley and Lex, both carriers of cystic fibrosis, want to have a child. They have produced one viable embryo via *in vitro* fertilisation (IVF) and have had this embryo genetically tested. The result shows that the embryo has two copies of the cystic fibrosis allele, and so is destined to develop the disease. Since Lesley and Lex prefer to have a child without cystic fibrosis, they decide to subject the embryo to gene editing to replace the faulty alleles. The gene-edited embryo is implanted into Lesley's uterus, and nine months later a child, Larry, is born. Having no cystic fibrosis alleles, Larry will neither develop cystic fibrosis nor pass it on to any children he may go on to have.

Lesley and Lex employed gene editing to prevent a genetic disorder in their child. As we will henceforth say, they ‘edited out disease’. By contrast, consider now:

*Selected Barry.* Bellamy and Blair want to have a child. They have produced two viable embryos via IVF and plan to carry only one of these embryos to term; the other will be discarded. Since Bellamy and Blair are both carriers of cystic fibrosis, they have had the embryos genetically tested, and have discovered that one has two cystic fibrosis alleles, while the other has none. Bellamy and Blair prefer to have a child without cystic fibrosis, so they select the second embryo for implantation into Bellamy's uterus, and nine months later a child, Barry, is born. Having no cystic fibrosis alleles, Barry will neither develop cystic fibrosis nor pass it on to any children he may go on to have.

In this case, Bellamy and Blair choose to bring one possible future child into existence, rather than another, on the basis that the chosen child will not suffer from a particular genetic disease. As we will henceforth say, they ‘select against disease’.

Some have argued that editing out disease, as in *Edited Larry*, is more morally problematic than selecting against disease, as in *Selected Barry*, since it poses greater risks to the resulting child and his offspring (Collins 2015; Friedmann *et al.*^2015^; Lanphier *et al.*^2015^). However, others have argued that gene editing also has moral advantages over genetic selection. According to Christopher Gyngell and Julian Savulescu, for example, editing out disease is in at least one respect preferable to selecting against it: it benefits the future child, while selecting against disease does not (Gyngell & Savulescu 2017: 33; Julian Savulescu [personal communication]).^1^ Others may have the same thought in mind when they claim that editing out disease is ‘therapeutic’ or ‘curative’—both adjectives that could not plausibly be applied to selecting against disease.^2^

Defenders of this thought may argue that, in *Edited Larry*, editing out disease benefits Larry because, had such gene editing not occurred, Larry would have been born with cystic fibrosis; he would thus have been, in at least one way, worse off than he actually is. In this respect, editing out disease-causing genes in an embryo is similar to treating a disease in an existing person.

By contrast, it might be held that selecting against disease, as in *Selected Barry*, does not benefit the child who comes into existence as a result, since it is not the case that this child would otherwise have existed with the disease.^3^ Had Bellamy and Blair not selected against disease, then presumably they would have decided between their possible future children on some other basis, perhaps just randomly, and we can assume that either Barry would not have existed (e.g. because Bellamy and Blair would have implanted a different embryo), or would still have existed, without disease (e.g. because they would have implanted the same embryo, though not because it lacked genes for cystic fibrosis).

It is often assumed that, other things being equal, we have stronger moral reasons to benefit particular people than we do to bring about impersonal improvements (see, for example, Zohar 1995: 276–8; McMahan 1998: 473; Savulescu *et al.*^2006^). We do not commit ourselves to this view, but let us assume, for the sake of argument, that it is correct. One could then argue as follows:

1. Editing out disease benefits the future child
2. Selecting against disease does not benefit the future child, though it may produce an impersonal improvement
3. Other things being equal, we have stronger moral reasons to benefit particular people than to produce impersonal improvements
  Therefore
4. Other things being equal, we have stronger moral reasons to edit out disease than to select against disease.^4^

In this article, we challenge this argument—henceforth, the ‘benefit argument’. We argue (i) that the first premise fails to hold in relation to many likely future instances of editing out disease, and (ii) that restricting the scope of the premise to avoid this problem deprives the argument of much of its practical significance.^5^

## II. QUALIFICATIONS

Before we proceed to our critique of the benefit argument, we wish to offer some qualifications intended to present the benefit argument in its most favourable light (this section), and to defend it against a different critique (section 3).

The first set of qualifications concerns premise (1), according to which editing out disease benefits the future child. There will be cases in which editing out disease does not confer a benefit on the gene-edited child because either (i) the gene-edited embryo in fact never gives rise to a child, for example, because a miscarriage occurs before the child comes into existence, (ii) the editing procedure alters the numerical identity of the child to whom the embryo will give rise (we will return to consider this issue in more detail below), (iii) unusually, the genetic disorder would have increased rather than diminished the wellbeing of the child (disorders are not *always* detrimental to wellbeing), or (iv) the editing procedure has unintended effects that result in lower wellbeing for the child than she would have had without the procedure. Thus, (1) will need to be restricted to cases in which none of these circumstances obtain. We capture this restriction by revising (1) to

(1′). *If all goes according to plan*, editing out disease benefits the future child.

We stipulate that ‘all goes according to plan’ means simply that (i)-(iv) are all false.

Consider next premise (2), according to which selecting against disease does not benefit the child created as a result. Again, this requires qualification. We can imagine unusual scenarios in which the child would have been worse off, had the parents not selected against disease, and thus appears to receive a benefit as a result of the selection. For example, perhaps, had they not selected against disease, the parents in *Selected Barry* would have donated both embryos to other prospective parents who would have raised the resulting children in deprived circumstances.^6^ In the interests of charity, we assume that, in cases of selecting against disease, the child who comes into existence as a result of the selection procedure would either not otherwise have existed, or would have existed in precisely the same state as the state in which she in fact exists.

Further qualifications are also required, however. There are certain kinds of benefit that selecting against disease arguably *can* confer, even accepting the assumption that we have just introduced. The selected child may perhaps enjoy *non-comparative* benefits (Bykvist 2007; McMahan 2013). These are benefits that consist in being in a state that is in one respect good for you, not compared to some alternative scenario in which you might have been or were in, but in an absolute sense. In *Selected Barry*, Barry may receive non-comparative benefits simply by virtue of living a good life, or a life containing good elements.

Could selecting against disease also confer a *comparative* benefit? Here, things become more complicated. On the standard account of comparative benefit (which we will adopt for the moment, but revisit in section 5), event *E* confers a comparative benefit on an individual if and only if that individual is better off in the actual situation, in which *E* occurs, than she would have been had *E* not occurred. A comparative benefit of this kind can be existential or non-existential. *E* confers an *existential* comparative benefit on an individual if and only if that individual is better off in the actual situation, in which *E* occurs, than she would have been had *E* not occurred, *and the individual would never have existed had E not occurred*; never existing serves as the baseline situation from which benefit is measured (Persson 1995; Roberts 1998, 2003; see also Holtug 2001). Though the possibility of existential comparative benefits has been contested (Parfit 1984; Broome 1999; Bykvist 2007), it is at least arguable that in *Selected Barry*, Barry receives such a benefit—the benefit of existing rather than never existing—provided that his life contains net positive wellbeing.^7^

However, what selecting against disease arguably cannot confer, and editing out disease arguably can, is a *non-existential* comparative benefit. On the standard account of comparative benefit, *E* confers a non-existential comparative benefit on an individual if and only if that individual is better off in the actual situation, in which *E* occurs, than she would have been had *E* not occurred, *and the individual would still have existed at some point had E not occurred*; the baseline situation from which benefit is measured is an alternative state of existence, different in either its length or quality from the actual one.^8^ On this account, in *Selected Barry*, Barry does not receive such a benefit, since if Bellamy and Blair had not selected against disease, either Barry would never have existed, or he would have existed without disease (and would thus, we are assuming, have enjoyed the same level of wellbeing that he in fact enjoys).

With this distinction in hand, it may seem that we can rescue premise (2) by weakening it to

(2′). Selecting against disease does not confer a *non-existential comparative benefit* on the future child, though it may produce an impersonal improvement or confer a non-comparative benefit or existential comparative benefit on that child.

Combining the adjustments to both (1) and (2) made above leaves us with the following formulation of the benefit argument:

(1*) If all goes according to plan, editing out disease confers a non-existential comparative benefit on the future child
(2*) Selecting against disease does not confer a non-existential comparative benefit on the future child, though it may produce an impersonal improvement or confer a non-comparative benefit or existential comparative benefit on that child
(3*) Other things being equal, we have stronger reasons to confer non-existential comparative benefits on people than to produce impersonal improvements, confer non-comparative benefits on people, or confer existential comparative benefits on people

Therefore

(4*) Other things being equal, and if all will go according to plan, we have stronger moral reasons to edit out disease than to select against disease.

In what follows, we will adopt this formulation of the argument, though for ease of exposition we will use the unqualified term ‘benefit’ to refer to *non-existential comparative benefits*. We will continue to grant the third premise of the argument, i.e. (3*).

A final qualification concerns our understanding of what editing out disease and selecting against disease involve. We treat both as actions that are distinct and independent from the action of producing an embryo. Parents first produce an embryo, or a number of embryos, and then, separately, edit out disease, select against disease, or do neither. An alternative approach would regard both editing out disease and selecting against disease as composite actions: editing out disease involves producing an embryo and then editing it to remove the disease-causing genes, and selecting against disease involves producing a number of embryos and selecting between them based on genetic information about them. However, on this approach, the benefit argument clearly fails, since both editing out disease and selecting against disease affect whether the edited/selected child comes into existence.

## III. WHEN DOES THE CHILD COME INTO EXISTENCE?

A potential objection to the benefit argument points to a question on which we have so far remained silent: When does a child come into existence—before or after the gene editing or genetic selection takes place? In *Edited Larry*, for instance, is the embryo that is subjected to the editing procedure already Larry, or is it a precursor of Larry—an entity that will, at some point, give rise to Larry? The objection holds that, in seeking to answer this question, the proponent of the benefit argument faces a dilemma: either the embryo is already Larry, in which case the second premise (2*) of the benefit argument is false—selecting against disease will sometimes confer a benefit—or the embryo is not yet Larry, in which case the first premise (1*) of the benefit argument is false—editing out disease does not confer a benefit. Let us explain.

Suppose first that the embryo is already Larry. In that case, we should surely allow that the embryo in *Selected Barry* is also already Barry. Recall that in *Selected Barry*, Bellamy and Blair select an embryo with no cystic fibrosis alleles and carry that embryo to term, resulting in the birth of Barry. If the selected embryo is already Barry at the time that the selection takes place, then it seems that selecting against disease may benefit Barry by extending his life. Suppose that Barry goes on to live a good and healthy life to the age of 80. And suppose that, had they not selected against disease, Bellamy and Blair would have discarded this embryo and implanted another. Under these suppositions, selecting against disease benefits Barry by giving him a good and long life when he would otherwise have had a very short and presumably valueless—or almost valueless—life as an embryo before being discarded. This contradicts the second premise of the benefit argument, according to which genetic selection confers no benefit on the selected child.^9^

Suppose instead, then, that the embryo in *Edited Larry* is not already Larry, but merely a precursor of Larry. In that case, it may seem clear that editing out disease does not benefit Larry, and that the first premise of the benefit argument is therefore false. It may seem that if, at the time of the gene editing procedure, the child-to-be does not yet exist, the gene editing procedure simply determines what kind of child comes into existence (one with or one without a genetic disorder). Gene editing is just one way of performing genetic selection. By editing out disease, Lesley and Lex bring it about that Larry comes into existence rather than some alternative child—one with many of the same genes, but without cystic fibrosis.

We believe that a proponent of the benefit argument can escape this putative dilemma by advancing two plausible claims.

First, she can maintain that the embryo in cases like *Edited Larry* and *Selected Barry* is not yet the child to which it will give rise, thus escaping the first horn of the dilemma. Various arguments could be adduced in support of this claim. One such argument holds that, before the development of the primitive streak at around fifteen days post-fertilisation, the embryo does not have sufficient structural integrity to be an individual human being or person, and indeed could still form multiple individuals through twinning (or multiple early embryos could become one through fusion) (Persson 1995). Another claims that Larry is essentially a person and denies that the early embryo can be a person, for example on the basis that it lacks consciousness (McMahan 2003).

Second, in order to escape the second horn of the dilemma, she can maintain that, even though editing out disease occurs before the child comes into existence, it does not affect which child *will* exist, so it can still benefit the child who does in fact come into existence. In support of this claim, she can note that genetic changes brought about before a child comes into existence often seem not to affect which child comes into being.^10^

An example may help to motivate this second claim. Imagine that a particular oocyte, *O*, is fertilised by a particular sperm cell, *S*, giving rise to a child called Duncan. Surely neither *O* nor *S* is identical to Duncan. Still, it is plausible that certain changes to either *O* or *S* could have affected the characteristics of the future child that would have been created, but without affecting whether that child was Duncan. This is most plausible for changes in genes that only manifest themselves phenotypically later in life and have only trivial effects. Suppose we change a gene in *O* and that the only effect of this change is to make it the case that the resulting person will, at the age of 70, experience a tingling in his left pinkie for five minutes. It seems implausible to suppose that this change affects the numerical identity of the child to whom *O* and *S* give rise.

It is, however, also plausible that genetic changes that occur before a child comes into existence can preserve numerical identity even when the genetic change causes more significant and earlier phenotypic effects, such as whether or not the child develops a devastating disease *during childhood*.^11^ Suppose an embryo will give rise to a child who can be expected, at age three, to develop a serious genetic cardiac condition. However, suppose that before this child comes into existence, a scientist discovers a means of blocking the development of this condition by regularly administering a drug to the early embryo: this drug binds to the causative gene and blocks its effects on cardiac development. It seems implausible that giving this drug to the early embryo, and thereby preventing the development of the cardiac condition, affects the numerical identity of the child that will exist. Moreover, this seems implausible even if one holds that the child does not exist at the time the drug is given. This at least casts doubt on the suggestion that editing out the causative genes *would* affect numerical identity.^12^

We think, then, that it is plausible both that embryos subjected to gene editing are not yet the child to which they will give rise, and that editing out disease does not alter the numerical identity of the child to which they will give rise. In what follows, we will simply assume that this is so. This allows the proponent of the benefit argument to escape the dilemma set out above. She can maintain that editing out disease confers a non-existential, comparative benefit on the gene-edited child, even while conceding that this child does not yet exist. However, we think that the proponent of the benefit argument faces a different and more serious problem. It is to this problem that we now turn.

## IV. WHAT WOULD HAVE HAPPENED?

Let us return to *Edited Larry*, in which Lesley and Lex edited the cystic fibrosis alleles out of the embryo from which Larry developed. The proponent of the benefit argument, who believes that editing out disease benefits Larry, envisions that, had the parents not edited out disease, Larry would have had cystic fibrosis. Thus, Larry would have been in one respect worse off than he in fact is. This is the counterfactual scenario that results from simply removing the action under consideration (editing out disease) from the actual scenario, while keeping all of the parents’ actual past and future actions fixed (they create the same embryo and have that embryo implanted into Lesley's uterus, Lesley still carries the embryo to term, and so on). But why think that this is what would have happened had the parents not edited out disease?^13^ Suppose that Lesley and Lex planned to use gene editing to ensure their child would not develop cystic fibrosis, but at the last moment they became worried about the risks of gene editing and cancelled the procedure. Absent any further information about Lesley and Lex, it is doubtful that they would then have decided to have the cystic fibrosis-affected embryo implanted into Lesley's uterus. After all, it is natural to assume that Lesley and Lex (a) want to become parents, and (b) want their child to be free of cystic fibrosis, and having decided not to edit out disease, there will likely still be other reasonable means available to them for realising both of these goals. They could, for example, pursue a further round of IVF and then employ genetic selection, they could have a child with the assistance of a gamete donor or donor embryo, or they could adopt a child. It is plausible to assume that, if Lesley and Lex had not edited out disease, they would have opted for one of these alternatives, none of which would have resulted in Larry coming into existence.^14^ If this is right, then their actual action of editing out disease did not benefit Larry since, had this not occurred, Larry would never have existed.

None of this excludes the possibility that, in some cases, editing out disease *does* benefit the edited child, because the child would otherwise have existed and been afflicted by the disease. However, there will also be many cases in which editing out disease does not benefit the edited child, for the reasons we have just given, and these cases suffice to undermine the benefit argument, as formulated above. Recall that the first premise of the benefit argument holds that

(1*) If all goes according to plan, editing out disease confers a non-existential comparative benefit on the future child.

This is most naturally read as a general claim—editing out disease *invariably* confers a benefit—but as we have just seen, this general claim does not hold. Editing out disease fails to confer a benefit where the edited child would otherwise never have existed.

To rescue the benefit argument, we could restrict the scope of this premise. We could adjust it to

(1^#^) If all goes according to plan, editing out disease confers a non-existential comparative benefit on the future child, *provided that the parent(s) would in any case have brought the embryo to term.*

Which requires us also to weaken the conclusion of the argument to

(4^#^) Other things being equal, and if all will go according to plan, we have stronger moral reasons to edit out disease than to select against disease, *provided that the parent(s) would in any case have brought the embryo to term*.

However, this alteration substantially limits the practical significance of the benefit argument, since many—perhaps most—situations in which parents would consider editing out disease are ones in which, if the editing does not take place, the embryo will be discarded, so the restricted version of the benefit argument will not apply.

Moreover, restricting the scope of the benefit argument in the way we have just outlined also has a surprising implication that limits the practical significance of the argument even further. The implication becomes apparent when we consider when, exactly, we should expect the benefit argument to apply—in which circumstances will it be the case that the prospective parents would have brought the embryo to term had they not edited out disease. One relevant factor here is the severity of the genetic disorder whose ‘editing out’ is under consideration. There is reason to believe that the more serious the disorder, the more plausible it is that the parents would have discarded the afflicted embryo had they not employed gene editing, and would instead have pursued alternative means of having a child; presumably, prospective parents are generally more motivated to avoid severe genetic disorders in their child than less severe disorders, or, for example, carrier status.

Consider a modified version of *Edited Larry*. In the modified case, the embryo from which Larry developed had two alleles not for cystic fibrosis, but for Gilbert's syndrome, a common, mild and usually asymptomatic genetic disorder of the liver. As in the original version of the case, Lesley and Lex choose to subject the embryo to gene editing and as a result Larry neither develops Gilbert's syndrome, nor is a carrier of the condition.

In this scenario, it seems plausible that the relevant baseline counterfactual scenario for determining the presence of benefit is one in which Larry is born with Gilbert's syndrome. This is because, had Lesley and Lex not edited out the disease for some reason, it is plausible to assume that they would have decided to carry the (non-gene-edited) embryo to term anyway; Gilbert's syndrome would probably not be considered a serious enough disorder to outweigh the burden, risks, and possible moral costs associated with discarding the embryo and choosing an alternative way to have a child (e.g. by undergoing another round of IVF treatment and selecting against Gilbert's syndrome).

This leads to a surprising result: the more serious the disorder the prospective parents wish to avoid in their child, the less likely it is that editing out disease benefits the future child, and thus, if there are especially strong reasons to benefit particular people, the less likely it is that the benefit argument establishes a moral advantage for editing out disease over selecting against disease. The putative moral advantage for editing out disease is more likely to obtain for less serious disorders than for more serious ones.

This further diminishes the practical significance of the benefit argument, since the cases in which the benefit argument is most likely to apply—those in which the disorder is not serious—are also precisely the cases in which the benefit argument is least likely to be decisive in justifying the use of gene editing, since they are the cases in which other considerations are most likely to render gene editing impermissible. The main concern that has been raised about editing out disease in the literature (Collins 2015; Friedmann *et al.*^2015^; Lanphier *et al.*^2015^)—and a concern that is shared by at least one of those whose views inspired the benefit argument (Savulescu & Singer 2019)—adverts to its risks, either for the edited individual or her descendants. These risk-based concerns are most likely to be decisive when there is in any case a weak argument for editing out disease, since the disorder in question is not serious or even is trivial.

To recapitulate: we have argued that the benefit argument fails because, in some cases, editing out disease does not benefit the edited child, since that child would not otherwise have existed. We have also argued that restricting the argument to avoid this problem deprives it of much of its practical significance. It does so for two reasons. First, in many circumstances where gene editing is likely to be employed, the child would not otherwise have existed, so the restricted argument simply does not apply. Second, the cases in which the argument is most likely to apply are those in which gene editing is used to prevent a mild disorder, and these are precisely the cases in which the standard, risk-based objections to editing out disease are most likely to outweigh the moral advantages posited by the benefit argument.

In the remainder of the article, we will consider a possible objection to our argument thus far. The objection appeals to a problem with the standard account of comparative benefit that we have been deploying throughout.

## V. THE PRE-EMPTION PROBLEM

Our argument has relied, to this point, on the standard account of comparative benefit. On this account, an actual event *E* benefits an individual if and only if that individual is better off in the actual situation (in which *E* occurs) than she would have been had *E* not occurred. However, this account has recently come in for significant criticism. In this section, we consider how this criticism, if it is well-founded, might affect our critique.

The pre-eminent worry about the standard account of comparative benefit is that it seems to misclassify some harms as benefits. Consider:

*Hay Fever.* Mel and Marnie want a child and create an embryo through IVF. They have sadistic personalities and plan to use gene editing to create a child with a severe disease. However, they restrain themselves and use gene editing to inflict a less severe condition on their child; Mary is born with mild hay fever. Had they not inflicted this milder condition, they would have created a child with a severe disease such as cystic fibrosis.

The standard account of comparative benefit seems to imply that, in editing the embryo such that Mary has hay fever, Mel and Marnie benefit Mary, since she would have been worse off had they not done so. But this seems implausible. Surely the gene editing that resulted in her having hay fever did not benefit Mary—it harmed her. This suggests that, *contra* the standard account, the baseline scenario used to determine the presence of a non-existential comparative benefit cannot simply be what *would have happened* had the agent not performed the act in question. It might be argued, then, that in order to challenge the benefit argument, we cannot appeal to what the parents would have done in cases like *Edited Larry*, had they not edited out disease. In doing so, we would be appealing to precisely that feature of the standard account of comparative benefit that creates difficulties in cases like *Hay Fever*: the fact that what would have happened otherwise depends on the counterfactual actions of the agent whose actual action is under consideration.

The problem raised by *Hay Fever* is an instance of a more general problem faced by the standard account of comparative benefit, and the analogous account of comparative harm. (We henceforth use ‘the standard account’ to refer to the conjunction of the standard account of comparative benefit and the analogous account of comparative harm.) This problem has become known as the ‘pre-emption problem’. It arises also in cases that involve nothing comparable to gene editing. Consider:

*Punch:* Bob punches Frank in the face. Had Bob not punched Frank in the face he would have set him on fire.

Intuitively, Bob's punch has harmed, and not benefitted Frank, yet on the standard account, it has benefitted him (Norcross 2004).

Several responses to the pre-emption problem have been proposed. Some authors maintain that the standard account can withstand the problem, either because it does not have the implications that some find implausible, or because they are not as implausible as some have thought (Klocksiem 2012; Boonin 2014). Others have revised the standard account (Feit 2015; Hanna 2016). And others still have argued that none of these responses succeed (Johansson & Risberg 2017).

We cannot resolve the pre-emption problem here. Nor can we consider the implications of *all* credible responses to it for our argument. However, we can say *something* in reply. First, and most obviously, if the correct response to the pre-emption problem is simply to bite the bullet—to uphold the standard account of comparative harm and benefit and accept that there *is* a benefit in cases like *Hay Fever* and *Punch—*then clearly our critique of the benefit argument will stand. Second, and more interestingly, even if the pre-emption problem should lead us to deviate from the standard account, it is plausible that a version of our critique will stand. This is because some of the live alternative accounts also support a version of our critique.

In what follows, we will limit ourselves to considering three modified accounts of comparative harm and benefit: an intention-based account, a moralised account, and an option-based account. We will argue that each supports a version of our critique of the benefit argument. For the avoidance of doubt, we are not endorsing any of these modified accounts; all face significant problems and it may be that all are ultimately implausible. We are open to the possibility that a plausible account of comparative benefit and harm is yet to be found; the pre-emption problem is, we think, a hard problem. We are also open to the possibility that, unless and until such an account is found, we should either embrace the standard account as the least bad option (in which case our critique of the benefit argument stands) or suspend judgment on which account of comparative benefit and harm is correct (in which case we will need to suspend judgment on the benefit argument as well).

A further qualification: we will limit ourselves, in what follows, to cases in which the putatively beneficial or harmful event is a *voluntary action*. The pre-emption problem can also arise in relation to other types of event, but since our ultimate interest is in assessing a voluntary action—editing out disease—we can safely leave these cases aside.

### A. The Intention-Based Account

Consider first an *intention-based account* of comparative benefit and harm inspired by Hanna (2016, esp. at 266).^15^ On this account, to determine the presence of a benefit or harm, we compare the actual situation, in which some agent performs some action, to what would have happened had the agent performed neither that action *nor any other action of the same type*, where the type of an action is determined by the fundamental intentions that motivated it. This view may allow us to maintain that there is a harm in *Punch*, since Bob's punching Frank and Bob's setting Frank on fire are plausibly of the same type; both, presumably, are motivated by the same fundamental intention. Thus, the intention-based account will direct us to compare the actual situation to what would have happened had Bob neither punched Frank, nor set him on fire, nor performed any other action of the same type. This, plausibly, will be a situation in which Bob simply leaves Frank alone. Bob's punching Frank leaves him worse off than he would have been had Bob left Frank alone, thus Bob harms Frank.

Suppose that this account solves the pre-emption problem. Still, it won’t help the proponent of the benefit argument. In the cases of gene editing of interest to us here, the fundamental intention in play is plausibly the intention to avoid having a child with a serious genetic disorder. Thus, the relevant question is: what would the parents have done had they done nothing to avoid having a child with a serious genetic disorder?

Gene editing is performed on embryos that are outside the woman's body. Typically, as in *Edited Barry*, these embryos will have been created via IVF. We take it that in many cases of editing out disease, the parents will have created the embryo via IVF precisely because they wanted to avoid having a child with a serious genetic disorder, and IVF allows them to do this. They will have employed IVF in order to allow editing out disease or selecting against disease. In these cases, had the prospective parents done nothing to avoid having a child with a serious genetic disorder, they would not have employed IVF at all, so the particular embryo that they in fact created *in vitro* would never have existed. Perhaps they would instead have created an embryo through unassisted sexual reproduction. Thus, according to the intention-based account, the parents confer only an *existential* comparative benefit on the gene-edited child whom they bring into existence.

However, there will surely also be cases of editing out disease in which the IVF procedure that preceded the gene editing was *not* employed in order to avoid having a child with a serious genetic disorder. For example, there will be cases in which IVF was employed simply as a fertility treatment. Yet even in these cases, the prospective parents may have made decisions that *were* intended to allow gene editing to occur, and were motivated by an intention to avoid having a child with a serious genetic disorder. For example, the parents might have decided to attend one fertility clinic rather than another on the basis that the former provides gene editing services. In these cases, if the parents *had done nothing* to avoid having a child with a serious disorder, they would have undergone IVF in different circumstances, and probably at a different time, and different embryos would have been created. Compared to this baseline, editing out disease again confers only an existential comparative benefit, on the intention-based view.

There will, of course, be *some* cases in which, on the intention-based view, editing out disease *does* confer a non-existential comparative benefit, because, had the parents done nothing to avoid having a child with a disease, they would still have created the same embryo, and would then have implanted it and carried it to term without modifying it. But as the above scenarios show, there will also be many cases in which it does not confer such a benefit. Thus, the benefit argument will still need to be severely restricted in scope. A version of our critique will still stand.

But perhaps we have misinterpreted the intention-based account. We have been assuming that, on the intention-based account, to generate the appropriate counterfactual comparator situation, we must ‘excise’ all actions—past, present or future—performed from the same fundamental intention as the putatively harmful or beneficial action. But perhaps we ought to leave the past untouched. Perhaps, that is, we should compare the actual situation to what the agent would have done had she performed no actions from the same intention *now or hereafter*, but still performed all the actions that she actually performed from that intention in the past. Thus, in cases involving editing out disease, we should compare the actual situation to a counterfactual situation in which the prospective parents still create the embryo via IVF, but then, at the point at which they in fact employed the gene editing, they abruptly drop their intention of avoiding having a child with a serious disorder.

Would the parents then implant the embryo and carry it to term with the disease-causing gene? Possibly, but not necessarily. It depends on what intentions the parents would have had, had they not had the intention to avoid having a child with a serious disorder. The two most plausible candidates are that they would then have had the intention of having a child irrespective of its disease status, or that they would have had no intention to have a child (say, because their intention to have a child at all was contingent on an expectation that the child would probably be healthy). If the latter, they would not have implanted the embryo, and we again get the result that the gene editing confers only an existential counterfactual comparative benefit. So, again, a version of our critique stands; the benefit argument needs to be restricted in scope.

### B. The Moralised Account

Some of our discomfort about the implications of the standard account in the *Hay Fever* and *Punch* cases may derive from the fact that this account directs us to compare the actual situation to one in which the agent acts wrongly. This suggests another possible modification to the standard account: to determine the presence of a benefit or harm, we should compare the actual situation, in which some agent performs some action, not to what would otherwise have happened, but to what would have happened *had the agent performed no morally impermissible action*. Call this ‘the moralised account’.^16^ (As with the intention-based account, there is a question about whether, in generating the counterfactual comparator situation, we should hold the past fixed: should we excise past impermissible actions, or only present and future ones? In what follows, we will assume that the past should be held fixed, since this is the view most hospitable to the benefit argument.)

On the moralised account, it is plausible that Bob does not benefit, and in fact harms, Frank in *Punch*, since both punching Frank and setting him on fire are morally impermissible. Had Bob done nothing impermissible, it is plausible that he would simply have left Frank alone, or at least done nothing that would have resulted in significant suffering for Bob. Similar thoughts apply to *Hay Fever*. Both introducing the mild disease and introducing the more severe disease are morally impermissible. It is plausible that, had they acted permissibly, Mary's parents would have introduced no disease, and Mary would have been better off than in the actual scenario, in which she has hay fever. Employing the moralised account thus appears to generate the right verdict in the *Punch* and *Hay Fever* cases: it implies that there is harm in both cases.

Does the moralised account rescue the benefit argument from our critique? Perhaps it does if discarding embryos is morally impermissible—say, because it amounts to killing a person or potential person. On this view, the moralised account directs us to compare the case in which parents in fact edit out disease to what they would have done had they neither edited out disease, nor discarded the embryo. It is plausible that they would then have carried the embryo to term, resulting in a child afflicted with the disease, and thus worse off than in the actual situation.

However, this line of argument runs into at least two difficulties. First, and most obviously, many of us—including those whose views inspired the benefit argument—find it permissible to discard an embryo afflicted by a genetic disorder, or indeed, even a healthy embryo (see, for example, Savulescu 2002; Savulescu *et al.*^2006^). Many of us do not think that parents who embark on IVF but then decide not to implant any of the embryos act impermissibly. If we are right, the scenario in which the parents discard the embryo remains eligible to serve as the comparator situation.

Second, if a defender of the benefit argument did seek to defend the argument by appealing to the impermissibility of discarding embryos, this might rescue the argument, but at the cost of rendering it dialectically less interesting. If discarding embryos were impermissible, there would already be an objection to selecting against disease that does not apply to editing out disease: selecting against disease typically requires discarding multiple embryos and thereby requires impermissible actions. There would thus be no (or at least less) need to appeal to the benefit argument in order to establish a moral advantage for editing out disease.

### C. The Option-Based Account

The third alternative account of comparative harm that we wish to consider is inspired not by the literature on the pre-emption problem, but by that on gene editing. However, we consider it here because we think it can be supported by appealing to pre-emption cases.

Recall *Edited Larry*, in which Lesley and Lex edit out the cystic fibrosis genes from an embryo which is then carried to term, resulting in the birth of Larry. Suppose now that Lesley and Lex have strong religious objections to the discarding of human embryos. Thus, had they not edited the embryo that became Larry, they would still have brought him to term, and he would have gone on to develop cystic fibrosis. Call this variant of the case *Edited Larry (Pro-Life Parents)*.

On the standard account, editing out disease confers a non-existential comparative benefit in this case. This is one of the few cases in which our critique gets no purchase. But Tina Rulli (2019) has recently argued that, in cases like *Edited Larry (Pro-Life Parents)*, the parents do not benefit the future child, at least, not in the morally urgent way that ordinary treatments and cures benefit their recipients. True, the child would *in fact* have existed, and been worse off, had the gene editing not occurred. But the parents would have retained the *option* of preventing Larry from existing with the disease. As Rulli puts it, in cases like this, ‘[c]reation of a child with a high risk of genetic disease is not inevitable’, it is ‘still a matter of distinct and separate choice’ (2019: 1077). This, Rulli claims, prevents the benefit from being morally urgent in the way that standard therapeutic benefits are.

Rulli does not explicitly claim that there is *no* non-existential comparative benefit in cases like *Edited Larry (Pro-Life Parents)*. Rather, her claim is that there is no benefit *of the same morally urgent kind* as is present in typical cases of curing or treating a disease. However, there is some evidence that she may be tempted to make the stronger claim. Having rejected the view that there is, in cases like *Edited Larry (Pro-Life Parents)*, a benefit of the kind conferred by standard treatments and cures, she goes on to characterise the type of benefit conferred in such cases in existential terms. For example, she describes editing out disease as ‘a means for creating healthy lives’, and questions the moral urgency of pursuing it by casting doubt on the claim that there are urgent moral reasons ‘to create healthy people for their own sake’ (Rulli 2019: 1078). This suggests that Rulli *may* think that there is no non-existential comparative benefit in cases like *Edited Larry (Pro-Life Parents).* If she were indeed to think this, she could sustain this claim by adopting what we could call

*The option-based account*: an action confers a non-existential comparative benefit on a person if and only if, had the agent not performed the action, the agent would have had no option to prevent the person from existing in a worse-off state.

The option-based account not only sustains the claim that there is no non-existential comparative benefit in *Edited Larry (Pro-Life Parents)*, it also gets the right result in standard pre-emption cases, like *Punch* and *Hay Fever*. In *Punch*, the option-based account implies that there is no non-existential comparative benefit since, had Bob not punched Frank in the face, he could still have prevented Frank from being in a worse-off state: he could have refrained from setting him on fire. Similarly, in *Hay Fever*, Mary receives no non-existential comparative benefit, since had her parents not edited in the hay fever, they could still have prevented her from existing in a worse-off state; they could have prevented this by either not carrying the embryo to term, or carrying it to term without editing in any more serious condition.

Nevertheless, we doubt that the option-based account should be accepted. We worry that it will misclassify some benefits as non-benefits. Suppose that a doctor can prevent a patient from developing an untreatable painful disease by either giving Drug A now, or Drug B in ten days; if she does neither, the patient will develop the disease in twenty days. The doctor gives Drug A now. It seems to us plausible that this doctor benefits her patient by giving Drug A, even though it would not have been inevitable, from her perspective, that the patient would have developed the disease had she not given Drug A; she could have given Drug B instead. Alternatively, suppose that a pregnant woman takes folate supplements in the early days of pregnancy and thereby prevents a neural tube defect that would otherwise have developed in the embryo. It seems to us plausible (though perhaps not *obvious*) that the woman's taking folate may confer a non-existential comparative benefit on the future child even if she also has the option of aborting the pregnancy before the embryo develops to the point that it is identical with the future child—that is, before the future child comes into existence.^17^ In both of these cases, however, the option-based account implies that there is no non-existential comparative benefit.

Thus, while we think that Rulli has indeed identified a class of benefits that are typically particularly morally urgent, we are doubtful that her view can be further developed into a compelling general account of non-existential comparative benefits; we suspect the option-based account will have to be rejected. Still, it is worth noting that, if the option-based account *were* accepted, this would strengthen our critique of the benefit argument, since it would suggest that the range of cases in which editing out disease benefits the future child is even narrower than we argued above. On the option-based account, the future child receives a benefit only in rather unusual cases where, had the parents not edited out disease, they would have had no choice but to bring the embryo to term with the disease. These cases are likely to be very rare indeed.

## VI. CONCLUSION

In this article we have challenged the benefit argument, according to which editing out disease is in one respect morally preferable to selecting against disease: it benefits the future child. We first qualified the argument in order to present it in its most favourable light. We then defended it against a potential objection, according to which either the embryo is not yet the child it will become, in which case editing out disease only confers an *existential* benefit, or, if the embryo is already identical with the future child, it does confer a non-existential benefit, but so does selecting against disease. Against this objection, we argued that one can at the same time deny that the embryo is the child it will become *and* deny that editing out disease affects the identity of the child who will exist. We then argued that the benefit argument does succumb to a different objection: there will be cases where the child would not have existed had the parents not edited out disease, and hence does not benefit from the editing. Moreover, we claimed that restricting the argument to avoid this problem deprives the argument of much of its practical significance. This is because (i) there will be many cases in which the child would not otherwise have existed and where the restricted argument simply does not apply, and (ii) the cases in which the argument is most likely to apply are those in which gene editing is used to prevent a mild disorder, and these are precisely the cases in which the standard, risk-based objections to editing out disease are likely to outweigh the moral advantages posited by the benefit argument. Finally, we considered how our critique of the benefit argument fares in the face of the pre-emption problem—a problem faced by the standard account of comparative benefit that we deployed in our critique. Without claiming to be able to resolve this problem, we argued that our critique—or a version of it—stands if either (i) the standard account survives the pre-emption problem, or (ii) the correct response to the pre-emption problem is to adopt any of the three alternative accounts that we considered: the intention-based account, the moralised account, or the option-based account.^18^

## Footnotes

1 Gyngell & Savulescu (2017: 33) write that ‘even when selection can be used to avoid disease, [germline gene editing] may provide a more desirable option. Selection prevents disease by changing who comes into existence; whereas gene editing ensures those who come into existence have the best shot of living a full life. Using [germline gene editing] to avoid disease thus seems more analogous to curing a disease than [genetic selection via pre-implantation genetic diagnosis]’. The implicit claim here, we take it, is that germline gene editing is preferable to genetic selection for the reason that, like curing a disease, it *benefits* the gene-edited individual; genetic selection, by contrast, confers only impersonal benefits. Gyngell *et al.* (2017: 501) make the role played by benefit explicit, but, at the urging of one of the present authors, introduced a qualification to their claim to avoid the problem raised in the present article. There, they write that ‘genetic selection replaces one individual with a disease with a healthy individual. It does not benefit those with disease. Its benefits are impersonal. [Germline gene editing] on the other hand could provide benefits to individuals who would otherwise be born with genetic disorders—it could cure their disorders. *In cases where the embryo will in any case be brought to term*, and in the absence of [germline gene editing] would be afflicted by disease, its benefits are arguably person-affecting. It is plausible that person-affecting benefits are more important than impersonal benefits’ (our emphasis). In this article, we explain the need for the italicized qualification.

2 Such claims are frequently made in both scholarly and popular venues. For a summary, see Rulli (2019: 1075).

3 We will qualify this view in section 2, below.

4 Similar arguments are advanced by Cavaliere (2018) and Wrigley *et al.* (2015). Cavaliere writes that pre-implantation genetic diagnosis (PGD) ‘is a contested practice as its scopes are not therapeutic (i.e. PGD does not treat embryos) but rather selective (i.e. PGD selects the embryos that should be transferred in utero.’ (2018: 218) She argues that gene editing has an advantage over genetic selection because it is (pre-emptively) therapeutic rather than selective, and is therefore able to evade some of the arguments against genetic selection. However, she does not claim that gene editing confers *benefits* that selection does not. Wrigley *et al.* (2015) advance an analogue of the benefit argument in the context of mitochondrial replacement therapy.

5 Two other recent discussions of the ethics of gene editing could be read as offering alternative critiques of the benefit argument. Rulli (2019) questions whether gene editing could confer benefits that are as morally urgent as ordinary treatments or cures. Sparrow (2021) argues that, in order to overcome scientific limitations, gene editing will, for the foreseeable future, need to be combined with genetic selection, and in a way that will prevent it from conferring non-existential comparative benefits (or harms) on the child. We discuss Rulli's argument in section 5.C below [and for fuller critiques of it, see Schaefer (2020); Palacios-González (2021)].

6 We thank Kasper Lippert-Rasmussen for pressing us to consider this sort of case.

7 Though compare Benatar (2008).

8 Note that our account of the distinction between existential and non-existential comparative benefits does not imply that, when one saves the life of someone with positive wellbeing, one confers only an existential comparative benefit. When one saves a person's life, one brings it about that the person continues to exist, when she otherwise would have ceased to exist. However, what makes a benefit existential is that, had the putatively beneficial action not been performed, the individual would *never* have existed. When one saves a person's life, the person would, in the absence of the intervention, still have existed prior to one's action, and we can employ that prior state of existence as the comparator, so any comparative benefit will be non-existential.

9 In response, it might be argued that *morally significant* Barry is not benefitted by his parents selecting against disease, since morally significant Barry only comes into existence subsequently. Thus, it might be claimed, the non-existential comparative benefit conferred on Barry through selecting against disease is morally irrelevant. We think this response is promising, but will not pursue it further since, as it happens, we think there is an easier route out of the dilemma, as outlined below.

10 For a similar view, see Elliot (1993: 31) who argues that whether gene therapy disrupts identity will depend on the magnitude of the substitutions and the degree of informational alteration involved. Note, however, that Elliot appears here to be speaking of the identity of the altered embryo, not the resulting child. As Persson (1995) has argued, even if gene therapy does not affect the numerical identity of the embryo, it might still affect the numerical identity of the resulting child. However, Persson also thinks that most cases of gene therapy would not affect the future child's numerical identity (see esp. at 25).

11 For further discussion, see Zohar (1995); Elliot (1993); Persson (1995); and Bredenoord *et al.* (2011).

12 Of course, it might be argued that changes internal to the genome are more liable to affect numerical identity than other alterations with similar phenotypic effects.

13 The answer to the question ‘what would have happened had the parents not employed gene editing?’ will of course depend on what account of counterfactual conditionals one adopts. Everything we say on this matter is intended to be consistent with the most influential account, due to Stalnaker (1968) and Lewis (1973). On this account, what would have happened had the parents not employed gene editing is, roughly, what happens in the world, from among the possible worlds in which the parents do not edit out disease, that is closest to the actual world. For an account of how ‘closeness’ is to be understood, see Lewis (1979), esp. at 472.

14 There may be fewer alternative ways to have a healthy child when prospective parents will (almost) certainly pass on a heritable condition, for example, because one of them is homozygous for a dominant disorder, or both are heterozygous for a dominant condition or homozygous for a recessive condition. In such cases, selecting against disease may be a non-starter. Nevertheless, we suspect that many prospective parents in such cases will still look for alternative means to have an unaffected child (e.g. through the use of donor gametes, donor embryos, or adoption).

15 Note that Hanna's own account differs somewhat from the account we present here; for instance, it is an account only of harm, not benefit, and it applies only to cases of *free* action. However, the differences do not affect the arguments we will make.

16 One potential problem with this approach is that it could be circular. It is not clear how we can determine whether the action is morally permissible without invoking the concepts of benefit and harm.

17 Rulli (2019: 1080–1) argues that there are several morally significant differences between taking folate supplements and editing out disease, however none of these differences undermine the claim that these actions are alike in the respect that, on the option-based account, they confer non-existential comparative benefits. For a similar point, see Schaefer (2020: 383).

18 We thank, for their helpful comments on earlier versions of this article, Gabriel De Marco, Nils Holtug, Kasper Lippert-Rasmussen, Robert Ranisch, Julian Savulescu, Robert Sparrow, Frej Klem Thomsen, two anonymous reviewers for *Philosophical Quarterly*, and audiences at Roskilde University, Fondation Brocher (Geneva), and the Belgrade Center for the Study of Bioethics. We thank Tess Johnson for her research assistance. Katrien Devolder's research was funded in whole, or in part, by the Wellcome Trust [Grant number WT208189/Z/17/Z]. Thomas Douglas’ research was funded in whole, or in part, by the Uehiro Foundation on Ethics and Education. For the purpose of open access, the author has applied a CC BY public copyright licence to any Author Accepted Manuscript version arising from this submission.
